# Prediction of coronavirus 3C-like protease cleavage sites using machine-learning algorithms

**DOI:** 10.1016/j.virs.2022.04.006

**Published:** 2022-05-02

**Authors:** Huiting Chen, Zhaozhong Zhu, Ye Qiu, Xingyi Ge, Heping Zheng, Yousong Peng

**Affiliations:** Bioinformatics Center, College of Biology, Hunan Provincial Key Laboratory of Medical Virology, Hunan University, Changsha, 410082, China

**Keywords:** Coronavirus, 3C-like protease, Cleavage sites, Machine-learning algorithms, 3CLP webserver

## Abstract

The coronavirus 3C-like (3CL) protease, a cysteine protease, plays an important role in viral infection and immune escape. However, there is still a lack of effective tools for determining the cleavage sites of the 3CL protease. This study systematically investigated the diversity of the cleavage sites of the coronavirus 3CL protease on the viral polyprotein, and found that the cleavage motif were highly conserved for viruses in the genera of *Alphacoronavirus*, *Betacoronavirus* and *Gammacoronavirus.* Strong residue preferences were observed at the neighboring positions of the cleavage sites. A random forest (RF) model was built to predict the cleavage sites of the coronavirus 3CL protease based on the representation of residues in cleavage motifs by amino acid indexes, and the model achieved an AUC of 0.96 in cross-validations. The RF model was further tested on an independent test dataset which were composed of cleavage sites on 99 proteins from multiple coronavirus hosts. It achieved an AUC of 0.95 and predicted correctly 80% of the cleavage sites. Then, 1,352 human proteins were predicted to be cleaved by the 3CL protease by the RF model. These proteins were enriched in several GO terms related to the cytoskeleton, such as the microtubule, actin and tubulin. Finally, a webserver named 3CLP was built to predict the cleavage sites of the coronavirus 3CL protease based on the RF model. Overall, the study provides an effective tool for identifying cleavage sites of the 3CL protease and provides insights into the molecular mechanism underlying the pathogenicity of coronaviruses.

## Highlights

• A view of the diversity of the cleavage sites by the coronavirus 3CL protease.
• An accurate RF model for predicting the cleavage of coronavirus 3CL protease.
• A web-server named 3CLP for predicting the cleavage of coronavirus 3CL protease.
• The 3CLP is available at http://www.computationalbiology.cn/3CLPHost/home.html.
• 1352 human proteins were predicted to be cleaved by the coronavirus 3CL protease.

## 1 Introduction

The coronavirus is a kind of positive-sense single-stranded RNA viruses (Hartenian et al., 2020). It can be grouped into four genera including *Alphacoronavirus*, *Betacoronavirus*, *Gammacoronavirus*, and *Deltacoronavirus.* Seven coronaviruses have been reported to infect humans, including HCoV-NL63 and HCoV-229E (*Alphacoronavirus*), HCoV-OC43, HCoV-HKU1, severe acute respiratory syndrome coronavirus (SARS-CoV), Middle East respiratory syndrome coronavirus (MERS-CoV) and SARS-CoV-2 (*Betacoronavirus*) (Chen et al., 2020; Hartenian et al., 2020). Among them, SARS-CoV, MERS-CoV and SARS-CoV-2 are highly pathogenic and lethal (Gralinski et al., 2013; Chafekar and Fielding, 2018; Hu et al., 2021). Especially, the current pandemic caused by the SARS-CoV-2 has resulted in 476, 374, 234 human infections and 6,108,976 deaths globally as of March 25th, 2022 (WHO, 2022). How to effectively control the coronavirus is a great challenge for humans.

The coronavirus has a genome ranging from 27 to 32 ​kb in size (Cui et al., 2019). Most coronaviruses share a similar genomic structure which includes a polyprotein ORF1ab, four structural proteins (S, E, M and N), and a variable number of accessory proteins (Shang et al., 2021). The polyprotein ORF1ab could be cleaved into 16 non-structural proteins (NSPs) by the viral proteases which are NSP3/papain-like protease and NSP5/3C-like protease (Klemm et al., 2020). The 3C-like (3CL) protease, a typical cysteine protease, cleaves the ORF1ab at 11 sites and produces 12 NSPs (NSP5–NSP16) (Arya et al., 2021). These individual NSPs participate in multiple critical processes of viral infection such as the viral genome replication and transcription (Snijder et al., 2016; Arya et al., 2021). Besides, the 3CL protease can also cleave the host proteins (Wang et al., 2016; Zhu et al., 2017a, 2017b, 2020; Chen et al., 2019). A recent study by Moustaqil et al. found that the 3CL protease of SARS-CoV-2 could directly cleave TAB1 and NLRP12, which may provide a molecular mechanism for enhanced production of cytokines and inflammatory response observed in COVID-19 patients (Moustaqil et al., 2021). Due to the important role of 3CL protease in viral infection, it has been taken as a critical target for antiviral drug development (Anand et al., 2003; Fu et al., 2020; Vuong et al., 2020).

The cleavage sites of the 3CL protease are relatively conserved. Previous studies have shown that the first position in the upstream of the cleavage site, defined as P1 according to Schechter and Berger's study (Schechter and Berger, 1967), was highly conserved with the amino acid (AA) Q (Anand et al., 2003; Fang et al., 2010). Besides, other positions near the cleavage site also showed strong preferences to some AAs (Anand et al., 2003; Chuck et al., 2010). For example, P2, P3 and P4 preferred the high-hydrophobicity residues, positively charged residues, and small hydrophobic residues, respectively, while the downstream positions P1′ and P2′ both preferred small residues (Chuck et al., 2011). However, considering the large diversity of coronaviruses (Cui et al., 2019), there is still a lack of a systematic study towards the diversity of the cleavage sites by the coronavirus 3CL protease.

Lots of coronavirus polyproteins lack of annotations in the public databases due to a lack of effective tools for determining the cleavage sites on the polyprotein. Besides, only a few host proteins were experimentally determined to be cleaved by the viral 3CL protease (Wang et al., 2016; Zhu et al., 2017a, 2017b, 2020; Chen et al., 2019; Moustaqil et al., 2021; Pablos et al., 2021). It is in great need to develop more effective methods for determining the cleavage sites of the coronavirus 3CL protease. There are currently two kinds of computational methods for determining the cleavage sites of the virus protease. The first is the machine-learning methods (Singh and Su, 2016; Stanley et al., 2020). For example, Kiemer et al. developed a neural network model NetCorona to predict the cleavage sites of the coronavirus 3CL protease with high accuracy (Kiemer et al., 2004). Unfortunately, only 77 cleavage sites from seven full-length coronavirus genomes were used to train the model, which may lead to potential bias. The other is the homology-based method which infers the cleavage sites of polyproteins by sequence alignment to the reference sequences with known cleavage sites (Larsen et al., 2020). Although the homology-based method is accurate for sequences which are highly similar to reference sequences, it can be hardly applied for those with large diversification to reference sequences, and is unable to annotate the host proteins cleaved by the 3CL protease.

This work systematically investigated the diversity of the cleavage sites of 3CL protease in coronaviruses and built a random forest (RF) model for predicting the cleavage sites of the coronavirus 3CL protease with high accuracy. The RF model was further tested by the experimentally determined cleavage sites in several coronavirus host proteins; then, the RF model was used to predict the cleavage sites of the coronavirus 3CL protease on the human proteome; finally, a user-friendly online server named 3CLP was built to predict the cleavage sites of the coronavirus 3CL protease based on the RF model. The work would not only help understand the specificity of the coronavirus 3CL protease, but also facilitate the annotation of proteins cleaved by the coronavirus 3CL protease.

## 2 Materials and methods

### 2.1 The coronavirus cleavage sites

At least one polyprotein (ORF1ab) sequence with the known cleavage sites of the 3CL protease were obtained for 14 coronavirus species in three genera including *Alphacoronavirus*, *Betacoronavirus* and *Gammacoronavirus* from the NCBI RefSeq and protein databases on April 15th, 2021 (Supplementary Table S1). For each coronavirus species with the known cleavage sites on at least one polyprotein, the polyprotein sequences were obtained from the NCBI protein database and were aligned with MAFFT (version 7.427) (Katoh and Standley, 2013). Since the cleavage of polyproteins by 3CL protease is important for viral infection, the cleavage sites on polyproteins of the same viral species are hypothesized to be highly conserved and were obtained with the homology-based method (Larsen et al., 2020). A window of 20 AAs centered on each cleavage site was obtained from each sequence. Previous studies have shown that the P1 position was highly conserved with Q (Anand et al., 2003; Fang et al., 2010). Therefore, only the windows with Q in P1 were kept. The windows from viruses of the same genus were combined together and were de-duplicated. The number of unique windows in each genus was listed in Supplementary Table S2.

### 2.2 The data for modeling

Because positions P2–P4 (P1 was excluded because it was supposed to be completely conserved) and P1′–P2′ were more conserved than other positions (Supplementary Figure S1), the motifs containing residues in these positions were further extracted and were defined as the cleavage motifs. A total of 905 cleavage motifs were obtained from the genera of *Alphacoronavirus*, *Betacoronavirus* and *Gammacoronavirus.* They were then de-duplicated, which resulted in 265 unique cleavage motifs. They were taken as positive samples in the modeling. To obtain the negative samples, the Qs in polyprotein sequences of 14 coronavirus species mentioned above were identified except those in the cleavage sites; then, for each Q, a non-cleavage motif containing the neighboring three AAs in the upstream of Q and two AAs in the downstream of Q was built. A total of 6828 non-cleavage motifs were obtained. Based on the one-hot encoding, these non-cleavage motifs were grouped into 265 clusters by the k-means method using the module of sklearn.cluster in Python (version 3.7) (Pedregosa et al., 2011). One motif was randomly selected from each cluster, which led to 265 negative samples.

Then, the positive samples were encoded with the one-hot method, and were clustered into five groups by the k-means method. To ensure the balance of the positive and negative samples in the training and validation process, the negative samples were randomly separated into five groups to match the positive sample groups. The above processes were repeated five times and five datasets were generated. The size of each group in five datasets was listed in Table 1.

**Table 1:** The size of five groups in each dataset. The positive group and the corresponding negative group had the same size.

	Dataset1	Dataset2	Dataset3	Dataset4	Dataset5
	Pos/Neg	Pos/Neg	Pos/Neg	Pos/Neg	Pos/Neg
Group 1	99/99	38/38	48/48	78/78	58/58
Group 2	58/58	77/77	75/75	54/54	77/77
Group 3	30/30	52/52	39/39	79/79	79/79
Group 4	58/58	41/41	72/72	34/34	20/20
Group 5	20/20	57/57	31/31	20/20	31/31

### 2.3 The AA indexes

A total of 566 AA indexes were obtained from the AAindex database (version 9.2) on November 18th, 2020 (Kawashima et al., 2008).

### 2.4 Logo of sequences centered the cleavage sites

The logo of sequences centered the cleavage sites was generated with WebLogo 3 using the default parameters on April 16th, 2021 (Crooks et al., 2004).

### 2.5 Machine-learning modeling with random forest, support vector machine and naive bayes

Three machine-learning algorithms, random forest (RF), support vector machine (SVM) and naive bayes (10.13039/100004395NB), were used to predict the cleavage sites of the 3CL protease and were achieved using functions of RandomForestClassifier, svm.SVC, GaussianNB, respectively, with the default parameters in the package of sklearn in Python (version 3.7) (Pedregosa et al., 2011). Five times of five-fold cross-validations were used to evaluate the performance of the machine-learning algorithms. The AUC [Area Under the Receiver Operating Characteristic Curve (ROC)], AUPRC [Area Under the Precision-Recall Curve (PRC)], accuracy, sensitivity, specificity, false positive rate (FPR) and precision were used to evaluate the model performance. The AUC was calculated using the module of sklearn.metrics (Pedregosa et al., 2011); the AUPRC was calculated using the function of *pr.curve* in the R package of PRROC (Grau et al., 2015); the accuracy, sensitivity, specificity, FPR and precision were calculated based on the confusion matrix as the follows:

$$accuracy=\frac{TP+TN}{TP+TN+FP+FN}$$

$$sensitivity=\frac{TP}{TP+FN}$$

$$specificity=\frac{TN}{TN+FP}$$

$$FPR=\frac{FP}{TN+FP}$$

$$precision=\frac{TP}{TP+FP}$$

in which the TP, TN, FP and FN referred to true positive, true negative, false positive and false negative, respectively.

### 2.6 The principal component analysis (PCA)

The PCA of the AA indexes were achieved using the module of sklearn.decomposition in Python (version 3.7) (Pedregosa et al., 2011).

### 2.7 The workflow of the modeling process in predicting coronavirus 3CL protease cleavage sites

**Fig. 1:**
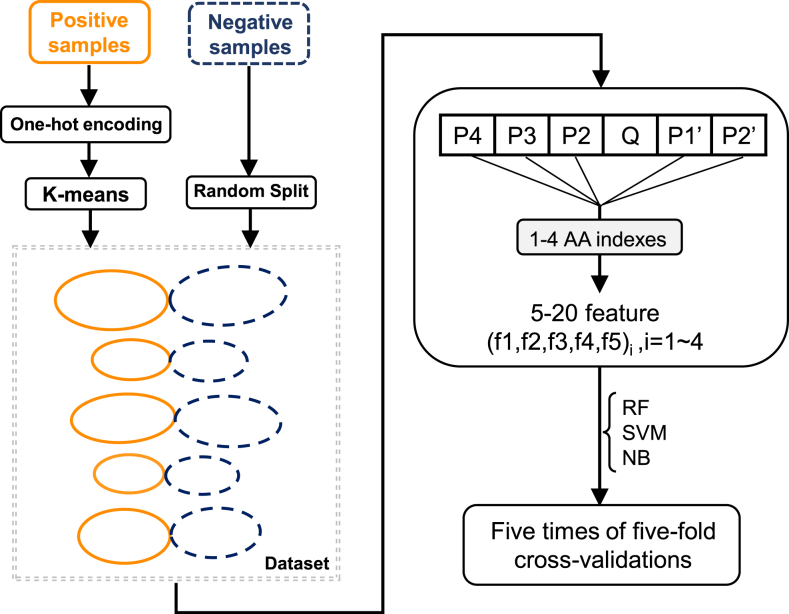
The work flow of the modeling process.

The work flow of the modeling process in predicting coronavirus 3CL protease cleavage sites was shown in Fig. 1. Firstly, the positive samples were clustered into five groups and the negative samples were randomly selected to match the positive samples. Then, the AAs in five positions (P2–P4, P1′–P2′) on the positive or negative samples were encoded by one to four AA indexes, which led to five to twenty features for each sample. For example, when using one AA index in AA encoding, the AAs in five positions were transformed into a numeric vector of length 5 (f1, f2, f3, f4, f5). Then, five times of five-fold cross-validations were used to evaluate the performance of three machine-learning algorithms, i.e., RF, SVM and NB, and were also used to select the number of AA indexes used in the modeling (see the texts in the Results section for details).

### 2.8 The human proteome

The human proteome was obtained from the SwissProt database in UniProt on June 29th, 2021. The protein sequences of 20,386 human proteins were obtained.

### 2.9 Functional enrichment analysis of human genes

The KEGG pathway and GO enrichment analysis was conducted with functions of enrichKEGG and enrichGO in the package clusterProfiler (version 3.18.1) in R (version 4.0.5) (Yu et al., 2012). All the KEGG pathways and GO terms with q-values less than 0.05 were considered as significant enrichment.

### 2.10 Statistical analysis

All the statistical analyses in this study were conducted in R (version 4.0.5). The Wilcoxon rank-sum test was used to compare the sample means in this study and was conducted with the function of *wilcox.test()* in R.

## 3 Results

### 3.1 The diversity of cleavage sites of the coronavirus 3CL protease

**Fig. 2:**
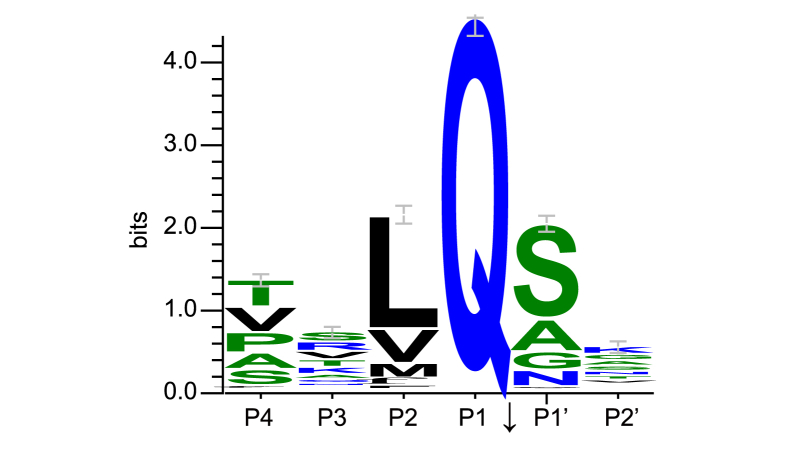
The logo for sequences around the cleavage sites of the coronavirus 3CL protease. The color of AAs refers to the hydrophobicity level, with the hydrophilic AAs (RKDENQ) colored in blue, the neutral AAs (SGHTAP) colored in green and the hydrophobic AAs (YVMCLFIW) colored in black. The overall height of the stack indicates the sequence conservation at that position, while the height of AAs within the stack indicates the relative frequency of each AA at that position.

The 3CL protease has 11 cleavage sites on the polyprotein ORF1ab of coronaviruses (Snijder et al., 2016). The cleavage sites of 3CL protease on polyproteins were obtained from 14 coronavirus species in three genera including *Alphacoronavirus*, *Betacoronavirus* and *Gammacoronavirus*. The logos of sequences around the cleavage sites for three genera (Fig. 2 and Supplementary Figure S1) showed similar residue conservation levels and residue preferences. Besides the P1, the P2, P1′ and P4 were the most conserved sites for all three genera. On the position of P2, the AAs of L, M and V were most conserved; on the P1′, the AAs of S, A, G and N were most conserved; on the P4, the AAs of T, V, P, A, and S were most conserved. When combined together, the P1–P4 and P1′–P2′ were more conserved than other positions (Supplementary Figure S1). Because the P1 was supposed to be completely conserved, the motif containing the P2–P4 and P1′–P2′ (defined as the cleavage motif) was kept for further analysis (Fig. 2).

### 3.2 Establishment of machine-learning models for predicting the cleavage sites of the coronavirus 3CL protease

**Fig. 3:**
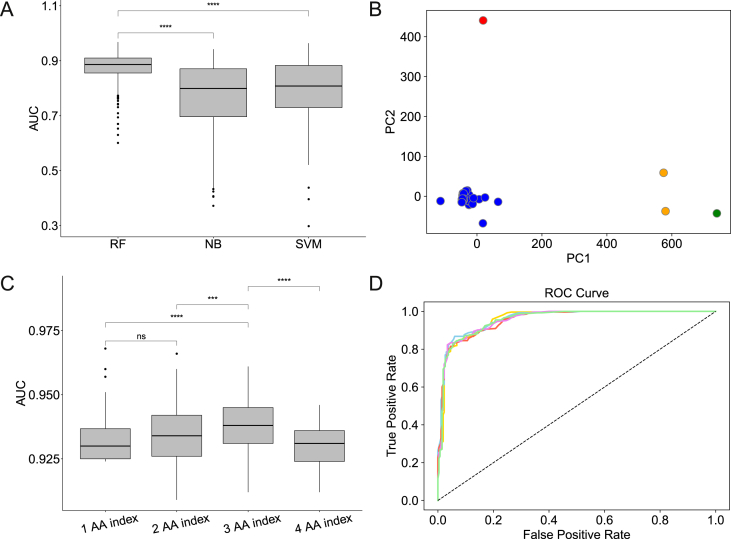
Prediction of cleavage sites of 3CL protease with the machine-learning algorithms. A Comparison of model performances with random forest (RF), and naive bayes (NB) and support vector machine (SVM) algorithms when using one AA index. **B** Visualization of the first two components of the top 10% AA indexes in the principal component analysis. These AA indexes were clustered into four clusters which were shown in different colors. **C** Comparison of AUCs of RF models with one to four AA indexes. For models with one AA index, only those built using the top 10% AA indexes were used; **D** The ROC and model performances of the best RF model using three AA indexes. The ROCs of the RF model in five times of five-fold cross-validations were shown in different colors. ∗∗∗, *P*-value < 0.001; ∗∗∗∗, *P*-value < 0.0001; ns, not significant. AUC, area under the ROC; ROC, the receiver operating characteristic curve; AA, amino acid.

A total of 265 cleavage motifs (positive samples) and equal number of non-cleavage motifs (negative samples, see Materials and methods) were obtained to build the machine-learning model for predicting the cleavage sites of the coronavirus 3CL protease. Three machine-learning algorithms including the RF, SVM and NB were used to build the model for predicting the cleavage sites of the 3CL protease, and a strict testing strategy of five times of five-fold cross-validations based on k-means clustering of the datasets (Table 1) was used to evaluate and compare the predictive performance of the algorithms. When using one AA index in the modeling, there were a total of 566 models for each algorithm. The RF models had a median AUC of 0.88, which were significantly higher than those of both the SVM and NB models (Fig. 3A). Therefore, the RF algorithm was used in the further modeling.

To improve the model performance, the top 10% AA indexes (58 AA indexes) (shown in Supplementary Table S3) in the RF models were analyzed with the PCA method. The first and second components were visualized in Fig. 3B. Four AA index clusters were obtained by the k-means clustering. To reduce the co-linearity of features in the modeling, combination of AA indexes was conducted by cluster. For example, when using two AA indexes in the modeling, two AA indexes were randomly selected from two different clusters independently. The RF models using all possible combinations of two, three and four AA indexes were built and evaluated. As shown in Fig. 3C, the RF models with two AA indexes had higher AUCs than those with one AA index; the model performances were further improved when using three AA indexes; however, the model performances were decreased when using four AA indexes. Overall, the RF models using three AA indexes performed significantly better than those with one, two or four AA indexes. Therefore, the RF model which had the highest AUC (0.96) among all models using three AA indexes was selected (Fig. 3D). More specifically, the accuracy, sensitivity, precision of the model were 0.88, 0.80, and 0.96, respectively. The RF model used the AA indexes of MEEJ800102, BIOV880102 and FASG760101, which referred to “the retention coefficient in high-pressure liquid chromatography”, “Information value for accessibility” and “Molecular weight”, respectively.

### 3.3 Validation and comparison of machine-learning models in predicting the cleavage sites of the coronavirus 3CL protease

**Fig. 4:**
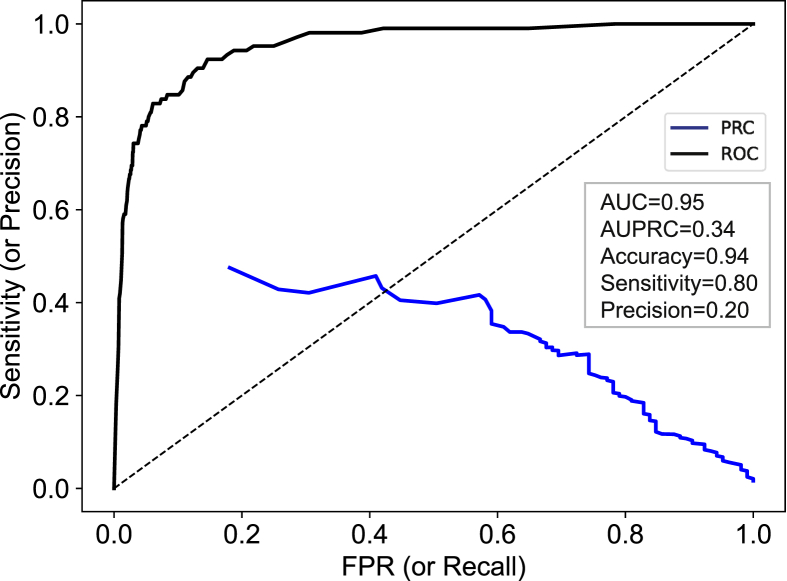
The receiver operating characteristic curve (ROC) (black) and precision-recall curve (PRC) (blue) of the random forest model on the test dataset. The accuracy, sensitivity and precision were shown when taking 0.50 as the cutoff for determining the predicted positive sample. AUC, the area under the ROC; AUPRC, the area under the PRC; FPR, false positive rate.

To test the RF model in prediction of the cleavage sites of the coronavirus 3CL protease, an independent test dataset derived from host proteins was manually curated from literatures (Supplementary Table S4). It contained 105 experimentally validated cleavage sites on 99 proteins from human, cat, pig and mouse. Except the AA of Qs in these cleavage sites, a total of 6,326 Qs in these proteins were hypothesized to constitute the non-cleavage sites. The RF model was tested and evaluated on the test dataset. It achieved an AUC of 0.95 (Fig. 4). A total of 84 experimentally validated cleavage sites were correctly predicted by the RF model. The prediction accuracy, sensitivity and precision were 0.94, 0.80 and 0.20, respectively (taking the default cutoff of 0.50 for determining the predicted positive sample) (Supplementary Table S5). Increasing the cutoff can increase the prediction precision and decrease the sensitivity (Fig. 4). For example, when taking 0.90 as the cutoff, the prediction precision was increased to 0.38, while the sensitivity was decreased to 0.59; when taking 0.99 as the cutoff, the RF model achieved the highest prediction precision (0.48) and the lowest sensitivity (0.18) (Supplementary Table S5).

Previous study by Kiemer et al. developed an artificial neural network model named NetCorona for predicting the cleavage sites of the coronavirus 3CL protease (Kiemer et al., 2004). For comparison, NetCorona was also evaluated on the test dataset using the related webserver which is available at https://services.healthtech.dtu.dk/service.php?NetCorona-1.0. It achieved a similar AUC with the RF model (AUC ​= ​0.95, Supplementary Figure S2). However, the AUPRC of NetCorona was smaller than that of the RF model (0.32 *vs* 0.34) (Supplementary Figure S2). When the sensitivity was in the range of 0.4–0.6, the prediction precision of both models were relatively stable, and the prediction precision of the RF model was 0.1 higher than that of the NetCorona (Supplementary Figure S2).

### 3.4 Application of the RF model in predicting the cleavage sites of the coronavirus 3CL protease on human proteins

**Fig. 5:**
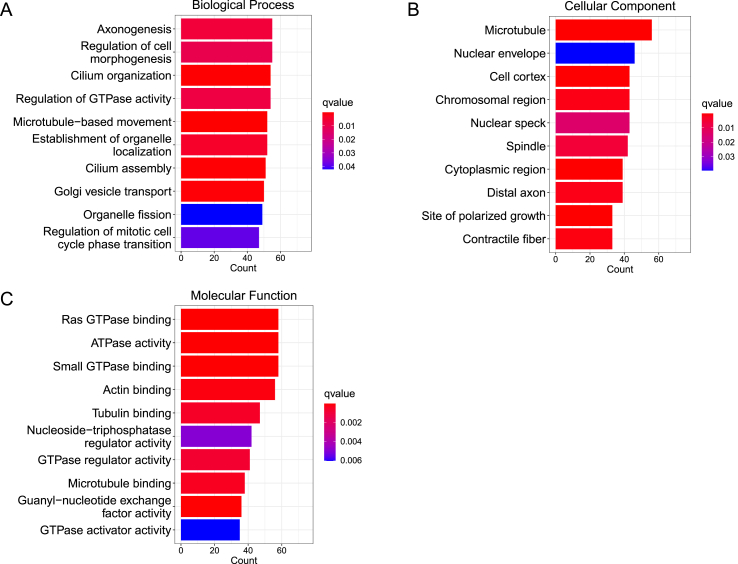
The functional enrichment analysis of the human proteins which were predicted to be cleaved by the coronavirus 3CL protease. Only top ten most enriched GO terms were shown. For more results, see Supplementary Table S6. **A**–**C** refer to the GO enrichment analysis in the domain of Biological Process, Cellular Component, Molecular Function, respectively.

Then, the RF model was used to predict the potential cleavage sites on human proteins by the coronavirus 3CL protease. To increase the prediction precision of the RF model, the cutoff for determining the positive was set to be 0.99 (Supplementary Table S5). A total of 1,352 human proteins were predicted to be cleaved by the coronavirus 3CL protease with 1,511 cleavage sites. Most of human proteins had only one predicted cleavage sites. Some proteins had more than three cleavage sites, such as the Golgin subfamily A member 3 (UniProtKB: Q08378). The GO enrichment analysis of the human proteins which were predicted to be cleaved by the coronavirus 3CL protease showed that in the domain of biological process, they were enriched in processes of organization, assembly, movement, localization, and so on (Fig. 5A and Supplementary Table S6); in the domain of cellular component, they were enriched in multiple locations such as microtubule, nuclear, cell cortex, spindle, and so on (Fig. 5B and Supplementary Table S6); in the domain of molecular function, they were enriched in binding, ATPase and GTPase activity, and so on (Fig. 5C and Supplementary Table S6). The KEGG enrichment analysis showed that these proteins were only enriched in two pathways including “Salmonella infection” and “Lysine degradation” (Supplementary Table S6).

### 3.5 Construction of the online server 3CLP

To facilitate the usage of the RF model mentioned above, a user-friendly online server named 3CLP was built for predicting the cleavage sites of the coronavirus 3CL protease. The input of 3CLP is the protein sequences of either viral or host proteins in the FASTA format; the prediction process would take several minutes depending on the number of protein sequences inputted; the outputs of 3CLP are the positions of the predicted cleavage sites, the motifs around the cleavage sites, and the score of the predicted cleavage sites which range from 0 to 1. The 3CLP is freely available to users without registration. The URL of 3CLP is http://www.computationalbiology.cn/3CLPHost/home.html.

## 4 Discussion

This work systematically investigated the diversity of the cleavage sites of coronavirus 3CL protease on the polyprotein and found that the cleavage sites were highly conserved in multiple genera of the coronavirus. The AA preference at neighboring positions of the cleavage sites of the 3CL protease were similar to that reported in previous studies. For example, hydrophobic and small AAs were preferred at the P2 and P1′ position, respectively (Chuck et al., 2011). This preference enabled us to build the computational models of predicting the cleavage site based on the AA indexes instead of the AA identity.

The machine-learning-based methods and the homology-based methods have been developed to predict the cleavage sites of the coronavirus 3CL protease. Compared to the homology-based methods, the machine-learning-based methods can be used to predict the potential cleavage sites on host proteins, facilitating the studies of the virus-host interactions in viral infection. In this study, we used a RF algorithm to predict the cleavage sites of coronavirus 3CL protease. The RF algorithm is a robust machine learning algorithm that can be used for a variety of tasks including regression and classification. It was extensively used in coronavirus research such as infection risk prediction (Qiang et al., 2020), disease diagnosis (Rosado et al., 2021), origin identification (El Boujnouni et al., 2021), drug development (Gupta and Mohanty, 2021), and so on. The RF model developed here used the cleavage sites on polyproteins from 14 coronaviruses for modeling which were more than three times to that used in Kiemer's study (Kiemer et al., 2004). Besides, our study used a very strict testing strategy by separating the dataset using the clustering method (Lu et al., 2021), which could reflect the ability of the model in predicting cleavage sites on polyproteins of novel viruses or host proteins. In the independent testing on host proteins, the RF model predicted the cleavage sites with higher precision and recall rate than the neural network model developed in Kiemer's study (Fig. 4). It could predict 80% of the cleavage sites correctly, suggesting its potential usage in predicting cleavage sites on host proteins.

Besides the cleavage on the viral polyproteins, the coronavirus 3CL protease can also cleave proteins involved in the host innate immune response such as NEMO and STAT2, thus evading the host immunity (Wang et al., 2016; Zhu et al., 2017a, 2017b). For example, the 3CL protease of both the feline infectious peritonitis virus (FIPV) and porcine epidemic diarrhea virus (PEDV) can interrupt the type I interferon (IFN) signaling by cleaving the NEMO, which led to the reduction of type I IFN (Wang et al., 2016; Chen et al., 2019). Our study predicted 1,511 potential cleavage sites in 1,352 human proteins. They were significantly enriched in several GO terms related to the cytoskeleton, such as the microtubule, actin, and tubulin. This suggests that the coronavirus infection may destroy the cytoskeleton by the viral 3CL protease, which may lead to several diseases related to cytoskeleton destruction such as the neurodegenerative diseases (Oberstadt et al., 2018; Kounakis and Tavernarakis, 2019). Previous studies have shown that a large proportion of COVID-19 patients have developed the neurological symptoms and some patients even developed the Parkinsonism after the SARS-CoV-2 infection (Acharya et al., 2020; Cohen et al., 2020; Dewanjee et al., 2021; Fearon and Fasano, 2021; Taquet et al., 2021). Some patients infected by the MERS-CoV and SARS-CoV also presented severe neurological symptoms or complications (Lau et al., 2004; Tsai et al., 2004; Xu et al., 2005; Arabi et al., 2015; Kim et al., 2017). Our study suggested that the neurological syndromes in patients infected by coronaviruses may be partly caused by the cleavage of critical proteins in nervous systems (such as actin and tubulin) by the viral 3CL protease. Further studies are needed to investigate the mechanism of neurological syndromes caused by the coronavirus.

There were some limitations in the study. Firstly, although the dataset was much larger than that used in previous studies, the dataset was still limited in size. The cleavage sites of the *Deltacoronavirus* were not included in the analysis. Nevertheless, the computational model developed here still showed high accuracy in both validations and testing, suggesting their potential usage in predicting the cleavage sites of the coronavirus 3CL protease. Secondly, the P1 position of the coronavirus 3C-like protease cleavage site was supposed to be highly conserved with Q, although there were a few cleavage sites with other residues in the P1 position (Pablos et al., 2021). More experimental efforts are needed to determine the AA specificity of the coronavirus 3CL protease cleavage sites.

## 5 Conclusions

This work systematically investigated the diversity of the cleavage sites of the coronavirus 3CL protease, which help understand the specificity of the protease. An RF model and the related server 3CLP for predicting the cleavage sites of the coronavirus 3CL protease was built with high accuracy and predicted a total of 1,352 human proteins which may be cleaved by the coronavirus 3CL protease. The work not only provides an effective tool for identifying the cleavage sites of the protease, but also provides insights into the molecular mechanism underlying the pathogenicity of coronaviruses.

## Data availability

All data used in the study were public available at 3CLP web-server which is available at http://www.computationalbiology.cn/3CLPHost/home.html.

## Ethics statement

This article does not contain any studies with human or animal subjects performed by any of the authors.

## Author contributions

Huiting Chen: data curation, formal analysis, methodology, validation, writing – original draft. Zhaozhong Zhu: investigation, formal analysis, software. Ye Qiu: supervision, writing – review & editing. Xingyi Ge: conceptualization, writing – review & editing. Heping Zheng: methodology, writing – review & editing. Yousong Peng: conceptualization, supervision, funding acquisition, methodology, writing – review & editing.

## Conflict of interest

The authors declare that they have no conflict of interest.
