# Supplementary material for: Prediction of coronavirus 3C-like protease cleavage sites using machine-learning algorithms
Source: Virol Sin. 2022 May 2;37(3):437–44. doi: 10.1016/j.virs.2022.04.006 (PMC9060714; doi:10.1016/j.virs.2022.04.006)
Supplement: Multimedia component 1 [file mmc1.docx]

***Virologica Sinica***

**Supplementary Data**

**Prediction of coronavirus 3C-like protease cleavage sites using machine-learning algorithms**

Huiting Chen ^a, 1^, Zhaozhong Zhu ^a, 1^, Ye Qiu ^a^, Xingyi Ge ^a^, Heping Zheng ^a^, Yousong Peng ^a,*^

^a^ Bioinformatics Center, College of Biology, Hunan Provincial Key Laboratory of Medical Virology, Hunan University, Changsha 410082, China

*Corresponding author.

*Email address*: [pys2013@hnu.edu.cn](mailto:pys2013@hnu.edu.cn) (Y. Peng)

ORCID: 0000-0002-5482-9506

^1^ Huiting Chen and Zhaozhong Zhu contributed equally to this work

**Supplementary Materials**


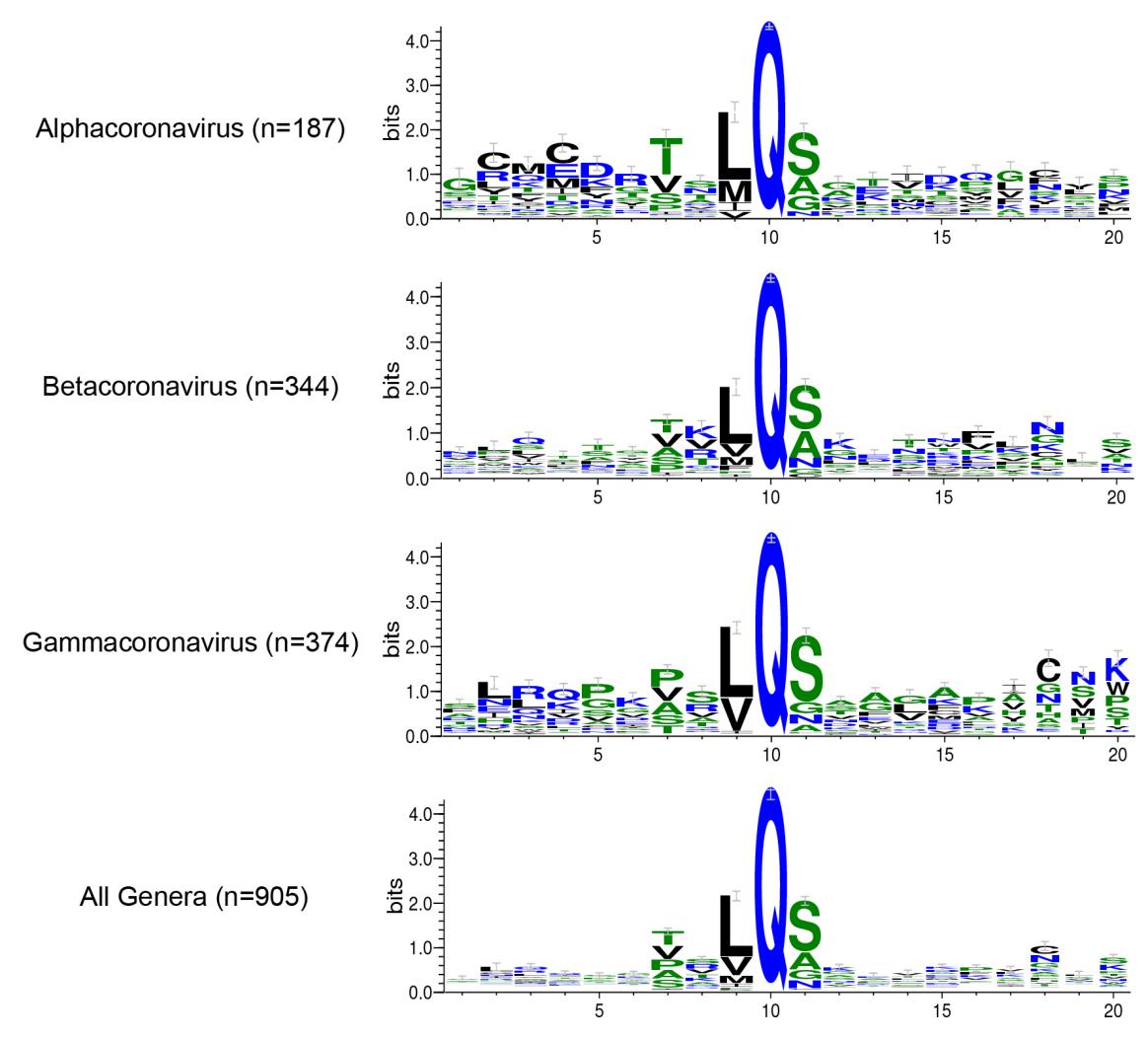


**Figure S1** The logo for the cleavage sites of coronavirus 3C-like protease in three coronavirus genus. The color of AAs refers to the hydrophobicity level, with the hydrophilic AAs (RKDENQ) colored in blue, the neutral AAs (SGHTAP) colored in green and the hydrophobic AAs (YVMCLFIW) colored in black. The overall height of the stack indicates the sequence conservation at that position, while the height of AAs within the stack indicates the relative frequency of each AA at that position.

**
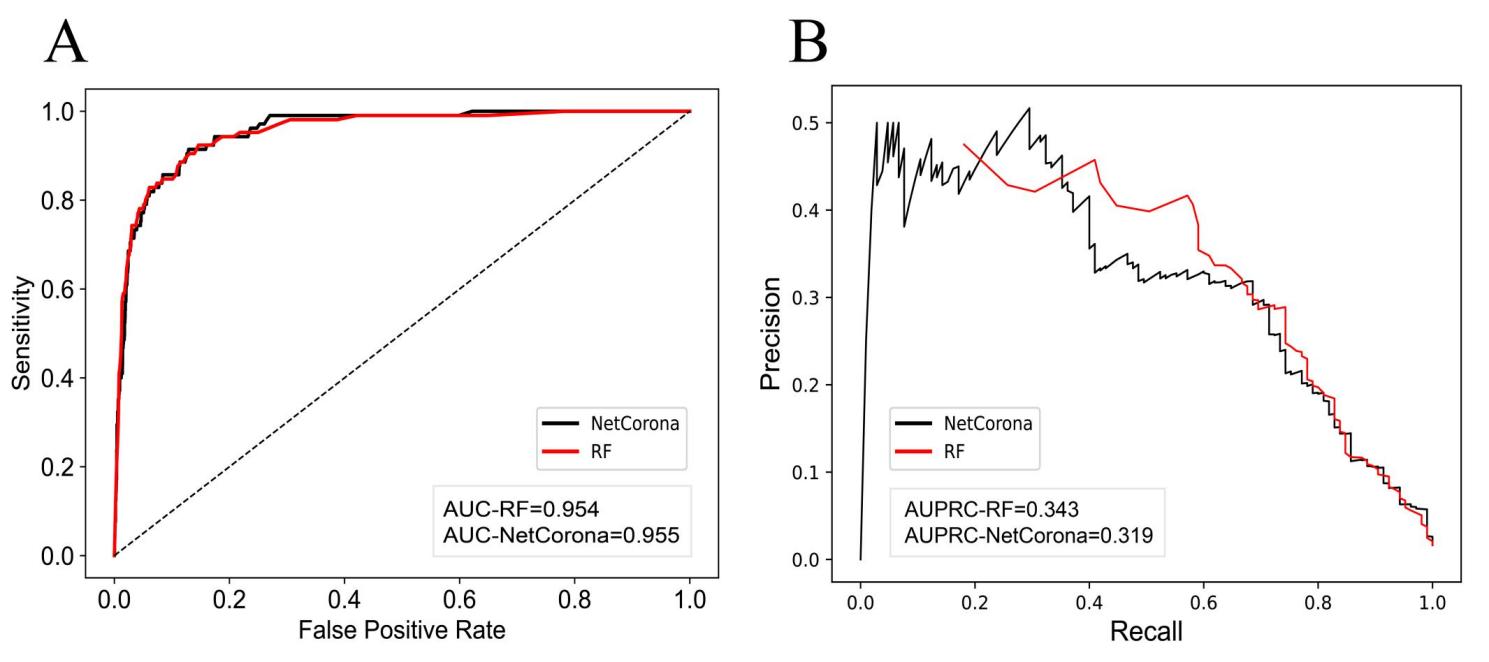
**

**Figure S2** The receiver operating characteristic curve (ROC) (**A**) and precision-recall curve (PRC) (**B**) of the random forest (RF) model (red) and the NetCorona (black) on the test dataset. AUC, the area under the ROC; AUPRC, the area under the PRC.

**Supplementary Tables**

**Table S1** Information of known cleavage sites in 14 coronavirus species.

| **Genus** | **Species** | **Version** | **Definition** | **Cleavage Information** |
| --- | --- | --- | --- | --- |
| Alphacoronavirus | Bat coronavirus CDPHE15 | YP_008439200.1 | replicase polyprotein 1ab  [Bat coronavirus CDPHE15/USA/2006] | 1..110=nsp1&111..896=nsp2&897..2558=nsp3&2559..3041=nsp4&3042..3344=3C-like proteinase nsp5&3345..3621=nsp6&3622..3704=nsp7&3705..3899=nsp8&3900..4007=nsp9&4008..4142=nsp10&4143..5070=nsp12&5071..5667=nsp13&5668..6186=nsp14&6187..6525=nsp15&6526..6825=nsp16 |
| Alphacoronavirus | Human coronavirus 229E | AGT21366.1 | replicase polyprotein 1ab  [Human coronavirus 229E] | 1..110=nsp1&111..897=nsp2&898..2488=nsp3&2489..2970=nsp4&2971..3272=3C-like proteinase; nsp5&3273..3551=nsp6&3552..3634=nsp7&3635..3829=nsp8&3830..3938=nsp9&3939..4073=nsp10&4074..5000=nsp12&5001..5597=nsp13&5598..6115=nsp14&6116..6463=nsp15&6464..6763=nsp16 |
| Alphacoronavirus | Human coronavirus NL63 | AGT51393.1 | replicase polyprotein 1ab  [Human coronavirus NL63] | 1..110=nsp1&111..898=nsp2&899..2461=nsp3&2462..2939=nsp4&2940..3242=3C-like proteinase nsp5&3243..3521=nsp6&3522..3604=nsp7&3605..3799=nsp8&3800..3908=nsp9&3909..4043=nsp10&4044..4970=nsp12&4971..5567=nsp13&5568..6085=nsp14&6086..6429=nsp15&6430..6729=nsp16 |
| Alphacoronavirus | Alphacoronavirus 1 | AGT52079.1 | ORF1ab polyprotein  [Feline coronavirus] | 1..110=nsp1&111..879=nsp2&880..2378=nsp3&2379..2868=nsp4&2869..3170=3C-like proteinase nsp5&3171..3464=nsp6&3465..3547=nsp7&3548..3742=nsp8&3743..3853=nsp9&3854..3988=nsp10&3989..4917=RNA-directed RNA polymerase nsp12&4918..5516=helicase nsp13&5517..6035=exoribonuclease nsp14&6036..6374=uridylate-specific endoribonuclease nsp15&6375..6674=2'-O-methyl transferase nsp16 |
| Betacoronavirus | Betacoronavirus 1 | YP_009555238.1 | ORF1ab polyprotein  [Human coronavirus OC43] | 1..246=leader protein&247..851=MHV p65-like protein&852..2750=PLpro&2751..3246=nsp4&3247..3549=3C-like proteinase&3550..3836=nsp6&3837..3925=nsp7&3926..4122=nsp8&4123..4232=nsp9&4233..4369=nsp10&4370..5297=RNA-dependent RNA polymerase&5298..5900=Hel&5901..6421=3' to 5' exonuclease&6422..6796=nsp15&6797..7095=2'-O-MTase |
| Betacoronavirus | Human coronavirus HKU1 | AGW27879.1 | replicase polyprotein 1ab  [Human coronavirus HKU1] | 1..222=nsp1&223..809=nsp2&810..2873=nsp3&2874..3369=nsp4&3370..3672=nsp5&3673..3959=nsp6&3960..4048=nsp7&4049..4245=nsp8&4246..4355=nsp9&4356..4492=nsp10&4493..5420=nsp12&5421..6023=nsp13&6024..6544=nsp14&6545..6918=nsp15&6919..7217=nsp16 |
| Betacoronavirus | Murine coronavirus | NP_045299.2 | ORF1ab polyprotein  [Murine hepatitis virus] | 1..247=leader protein&248..832=nsp2&833..3141=PLpro&3142..3333=nsp4&3334..3636=3C-like proteinase&3637..3923=nsp6&3924..4012=nsp7&4013..4209=nsp8&4210..4319=nsp9&4320..4456=nsp10&4457..5384=RNA-dependent RNA polymerase&5385..5984=Hel&5985..6505=3' to 5' exonuclease&6506..6879=NendoU&6880..7178=2'-O-MT |
| Betacoronavirus | Hedgehog coronavirus 1 | YP_009513008.1 | ORF1ab polyprotein  [Betacoronavirus Erinaceus/VMC/DEU/2012] | 1..200=nsp1&201..859=nsp2&860..2805=nsp3&2806..3310=nsp4&3311..3616=nsp5&3617..3908=nsp6&3909..3991=nsp7&3992..4190=nsp8&4191..4300=nsp9&4301..4440=nsp10&4441..5374=nsp12&4441..4454=nsp11&5375..5972=nsp13&5973..6496=nsp14&6497..6839=nsp15&6840..7150=nsp16 |
| Betacoronavirus | Middle East respiratory  syndrome-related coronavirus | AMO03400.1 | polyprotein 1ab  [Middle East respiratory syndrome-related coronavirus] | 1..193=nsp1&194..853=nsp2&854..2739=nsp3&2740..3247=nsp4&3248..3553=nsp5&3554..3845=nsp6&3846..3928=nsp7&3929..4127=nsp8&4128..4237=nsp9&4238..4377=nsp10&4378..5310=nsp12&5311..5908=nsp13&5909..6432=nsp14&6433..6775=nsp15&6776..7078=nsp16 |
| Betacoronavirus | Pipistrellus bat coronavirus HKU5 | YP_001039961.1 | ORF1ab polyprotein  [Pipistrellus bat coronavirus HKU5] | 1..195=nsp1&196..851=nsp2&852..2831=nsp3&2832..3338=nsp4&3339..3644=nsp5&3645..3936=nsp6&3937..4019=nsp7&4020..4218=nsp8&4219..4328=nsp9&4329..4467=nsp10&4468..5401=nsp12&5402..5999=nsp13&6000..6523=nsp14&6524..6874=nsp15&6875..7182=nsp16 |
| Betacoronavirus | Tylonycteris bat coronavirus HKU4 | YP_001039952.1 | ORF1ab polyprotein  [Tylonycteris bat coronavirus HKU4] | 1..195=nsp1&196..847=nsp2&848..2784=nsp3&2785..3291=nsp4&3292..3597=nsp5&3598..3889=nsp6&3890..3972=nsp7&3973..4171=nsp8&4172..4281=nsp9&4282..4420=nsp10&4421..5354=nsp12&5355..5952=nsp13&5953..6475=nsp14&6476..6817=nsp15&6818..7119=nsp16 |
| Betacoronavirus | Rousettus bat coronavirus HKU9 | YP_001039970.1 | orf1ab polyprotein  [Rousettus bat coronavirus HKU9] | 1..175=leader protein&176..772=nsp2&773..2609=PL-PRO&2610..3103=nsp4&3104..3409=3C-like proteinase&3410..3699=nsp6&3700..3782=nsp7&3783..3982=nsp8&3983..4094=nsp9&4095..4233=nsp10&4234..5165=Pol&5166..5766=Hel&5767..6296=ExoN&6297..6633=NendoU&6634..6930=2'-O-methyltransferase |
| Betacoronavirus | Severe acute respiratory  syndrome-related coronavirus | YP_009724389.1 | ORF1ab polyprotein  [Severe acute respiratory syndrome coronavirus 2] | 1..180=leader protein&181..818=nsp2&819..2763=nsp3&2764..3263=nsp4&3264..3569=3C-like proteinase&3570..3859=nsp6&3860..3942=nsp7&3943..4140=nsp8&4141..4253=nsp9&4254..4392=nsp10&4393..5324=RNA-dependent RNA polymerase&5325..5925=helicase&5926..6452=3'-to-5' exonuclease&6453..6798=endoRNAse&6799..7096=2'-O-ribose methyltransferase |
| Gammacoronavirus | Avian coronavirus | NP_066134.1 | ORF1ab polyprotein  [Infectious bronchitis virus] | 1..673=leader protein p87&674..2779=coronavirus nsp1 (HD1)&2780..3086=coronavirus nsp2 (3CL-Pro)&3087..3379=coronavirus nsp3 (HD2)&3380..3462=coronavirus nsp4&3463..3672=coronavirus nsp5&3673..3783=coronavirus nsp6&3784..3928=coronavirus nsp7 (GLF)&3929..4868=RNA-dependent RNA polymerase&4869..5468=coronavirus nsp10&5469..5989=coronavirus nsp11&5990..6327=coronavirus nsp12&6328..6629=coronavirus nsp13 |

**Table S2** The number of unique windows of 20 amino acid in each coronavirus species of three genera. The number in the brackets showed the number of unique windows.

| **Genus** | **Species** | **Number of windows** | **Total (unique)** |
| --- | --- | --- | --- |
| Alphacoronavirus | Alphacoronavirus 1 | 124 | 187 (187) |
|  | Bat coronavirus CDPHE15 | 10 |  |
|  | Human coronavirus 229E | 36 |  |
|  | Human coronavirus NL63 | 17 |  |
| Betacoronavirus | Betacoronavirus 1 | 94 | 350 (344) |
|  | Human coronavirus HKU1 | 16 |  |
|  | Murine coronavirus | 32 |  |
|  | Hedgehog coronavirus 1 | 17 |  |
|  | Middle East respiratory syndrome-related coronavirus | 84 |  |
|  | Pipistrellus bat coronavirus HKU5 | 18 |  |
|  | Tylonycteris bat coronavirus HKU4 | 16 |  |
|  | Rousettus bat coronavirus HKU9 | 33 |  |
|  | Severe acute respiratory syndrome-related coronavirus | 40 |  |
| Gammacoronavirus | Avian coronavirus | 374 | 374 (374) |

**Table S3** The top 10% AA indexes (58 AA indexes) and their AUCs in the random-forest modeling. The average of AUCs in five time five-fold cross-validations on the modeling dataset was shown.

| **Rank** | **AAindex** | **AUC** | **Rank** | **AAindex** | **AUC** | **Rank** | **AAindex** | **AUC** |
| --- | --- | --- | --- | --- | --- | --- | --- | --- |
| 1 | CORJ870104 | 0.968 | 21 | GARJ730101 | 0.933 | 41 | WERD780101 | 0.926 |
| 2 | GUYH850101 | 0.96 | 22 | CORJ870107 | 0.933 | 42 | MEEJ800101 | 0.925 |
| 3 | CORJ870103 | 0.957 | 23 | GEIM800107 | 0.932 | 43 | AVBF000101 | 0.925 |
| 4 | KRIW790102 | 0.951 | 24 | ROBB760105 | 0.932 | 44 | PONP930101 | 0.925 |
| 5 | PONP800106 | 0.95 | 25 | CHOP780202 | 0.932 | 45 | ROSG850101 | 0.925 |
| 6 | FUKS010104 | 0.949 | 26 | WEBA780101 | 0.931 | 46 | CHOC760101 | 0.925 |
| 7 | RADA880108 | 0.945 | 27 | FUKS010111 | 0.93 | 47 | NISK860101 | 0.925 |
| 8 | LEVM760107 | 0.941 | 28 | NAKH920108 | 0.93 | 48 | GUYH850105 | 0.925 |
| 9 | FUKS010102 | 0.941 | 29 | GUYH850102 | 0.93 | 49 | ISOY800107 | 0.925 |
| 10 | MEIH800102 | 0.938 | 30 | KARS160119 | 0.93 | 50 | FASG760101 | 0.925 |
| 11 | MEEJ800102 | 0.938 | 31 | BIOV880102 | 0.929 | 51 | LIFS790101 | 0.925 |
| 12 | ISOY800104 | 0.938 | 32 | LEVM760101 | 0.928 | 52 | LEVM780102 | 0.924 |
| 13 | CASG920101 | 0.938 | 33 | GEIM800106 | 0.928 | 53 | PONP800102 | 0.924 |
| 14 | KARS160112 | 0.937 | 34 | ZHOH040102 | 0.928 | 54 | BLAS910101 | 0.924 |
| 15 | EISD860103 | 0.937 | 35 | KUMS000102 | 0.927 | 55 | CHOP780208 | 0.924 |
| 16 | ZIMJ680102 | 0.936 | 36 | BASU050103 | 0.927 | 56 | OLSK800101 | 0.924 |
| 17 | FAUJ880101 | 0.936 | 37 | TANS770106 | 0.927 | 57 | NAKH900110 | 0.924 |
| 18 | RACS770102 | 0.935 | 38 | WOEC730101 | 0.926 | 58 | HOPT810101 | 0.924 |
| 19 | ZIMJ680103 | 0.934 | 39 | AVBF000109 | 0.926 |  |  |  |
| 20 | GEOR030105 | 0.934 | 40 | OOBM770104 | 0.926 |  |  |  |

**Table S4** Host proteins and the related cleavage sites of coronavirus 3C-like protease determined by experimental methods. They were used to test the ability of the RF model in predicting the cleavage sites of the coronavirus 3CL protease. For the cleavage sites from the study of Pablos et al. (PMID = 34672947), only the cleavage sites with Qs on the P1 position were used here since the RF model were built based on cleavage sites with Qs on the P1 position. “*”, not used in the testing.

| **PMID** | **Coronavirus Species** | **Host** | **Gene** | **Cleavage Site**  **(total = 105)** | **Accession** | **Number of Qs (total = 6431)** |
| --- | --- | --- | --- | --- | --- | --- |
| 31905881 | Feline Infectious Peritonitis Virus | Felis catus (domestic cat) | NEMO | Q132/Q205/Q231 | XP_019680064 | 56 |
| 28250121 | Porcine Deltacoronavirus | Sus scrofa (pig) | STAT2 | Q685/Q758 | BAA20332 | 64 |
| 32461317 | Porcine Deltacoronavirus | Sus scrofa (pig) | DCP1A | Q343 | I3LHS8 | 43 |
| 27984784 | Porcine Deltacoronavirus | Sus scrofa (pig) | NEMO | Q231 | NP_001106524 | 57 |
| 26656704 | Porcine Epidemic Diarrhea Virus |  |  |  |  |  |
| 33372854 | SARS-CoV-2 | Homo sapiens (human) | NLRP12 | Q238/Q938 | P59046 | 54 |
| 33372854 | SARS-CoV-2 | Mus musculus | NLRP12 | Q238 | NP_001028603 | 57 |
| 33372854 | SARS-CoV-2 | Homo sapiens (human) | TAB1 | Q132/Q444 | NP_006107 | 31 |
| 34672947 | SARS-CoV-2 | Homo sapiens (human) | MDC1 | Q1497 | Q14676 | 125 |
| 34672947 | SARS-CoV-2 | Homo sapiens (human) | SEPT9 | Q220 | Q9UHD8 | 24 |
| 34672947 | SARS-CoV-2 | Homo sapiens (human) | SRRM2 | Q1139/Q2150 | Q9UQ35 | 96 |
| 34672947 | SARS-CoV-2 | Homo sapiens (human) | SPTB2 | Q1871 | Q01082 | 153 |
| 34672947 | SARS-CoV-2 | Homo sapiens (human) | LARP1 | Q17 | Q6PKG0 | 55 |
| 34672947 | SARS-CoV-2 | Homo sapiens (human) | ATAD2 | Q954 | Q6PL18 | 66 |
| 34672947 | SARS-CoV-2 | Homo sapiens (human) | NU107 | Q35 | P57740 | 47 |
| 34672947 | SARS-CoV-2 | Homo sapiens (human) | RS21 | Q33 | P63220 | 2 |
| 34672947 | SARS-CoV-2 | Homo sapiens (human) | CREB1 | Q243 | P16220 | 36 |
| 34672947 | SARS-CoV-2 | Homo sapiens (human) | CLSPN | Q137 | Q9HAW4 | 62 |
| 34672947 | SARS-CoV-2 | Homo sapiens (human) | ZFY16 | Q445 | Q7Z3T8 | 66 |
| 34672947 | SARS-CoV-2 | Homo sapiens (human) | DYHC1 | Q1240 | Q14204 | 257 |
| 34672947 | SARS-CoV-2 | Homo sapiens (human) | FAF1 | Q53 | Q9UNN5 | 35 |
| 34672947 | SARS-CoV-2 | Homo sapiens (human) | RABE1 | Q65 | Q15276 | 88 |
| 34672947 | SARS-CoV-2 | Homo sapiens (human) | ZC3H4 | Q1003 | Q9UPT8 | 46 |
| 34672947 | SARS-CoV-2 | Homo sapiens (human) | WDR33 | Q1221 | Q9C0J8 | 107 |
| 34672947 | SARS-CoV-2 | Homo sapiens (human) | RPAP1 | Q236 | Q9BWH6 | 80 |
| 34672947 | SARS-CoV-2 | Homo sapiens (human) | IMA4 | Q78 | O00505 | 35 |
| 34672947 | SARS-CoV-2 | Homo sapiens (human) | NACAM | Q1913 | E9PAV3 | 53 |
| 34672947 | SARS-CoV-2 | Homo sapiens (human) | CLCB | Q92 | P09497 | 17 |
| 34672947 | SARS-CoV-2 | Homo sapiens (human) | PRC2A | Q174 | P48634 | 98 |
| 34672947 | SARS-CoV-2 | Homo sapiens (human) | SF3B2 | Q153 | Q13435 | 50 |
| 34672947 | SARS-CoV-2 | Homo sapiens (human) | IF4G2 | Q451 | P78344 | 62 |
| 34672947 | SARS-CoV-2 | Homo sapiens (human) | ARPC4 | Q17 | P59998 | 5 |
| 34672947 | SARS-CoV-2 | Homo sapiens (human) | BTAF1 | Q191 | O14981 | 109 |
| 34672947 | SARS-CoV-2 | Homo sapiens (human) | IF4G1 | Q37 | Q04637 | 84 |
| 34672947 | SARS-CoV-2 | Homo sapiens (human) | VIGLN | Q1014 | Q00341 | 64 |
| 34672947 | SARS-CoV-2 | Homo sapiens (human) | PSMD8 | Q89 | P48556 | 17 |
| 34672947 | SARS-CoV-2 | Homo sapiens (human) | SEPT6 | Q82 | Q14141 | 25 |
| 34672947 | SARS-CoV-2 | Homo sapiens (human) | SNUT1 | Q375 | O43290 | 39 |
| 34672947 | SARS-CoV-2 | Homo sapiens (human) | SPT6H | Q1639 | Q7KZ85 | 99 |
| 34672947 | SARS-CoV-2 | Homo sapiens (human) | GOGA2 | Q484 | Q08379 | 112 |
| 34672947 | SARS-CoV-2 | Homo sapiens (human) | PTBP1 | Q152 | P26599 | 29 |
| 34672947 | SARS-CoV-2 | Homo sapiens (human) | HNRPU | Q39 | Q00839 | 48 |
| 34672947 | SARS-CoV-2 | Homo sapiens (human) | TBB4A | Q8 | P04350 | 21 |
| 34672947 | SARS-CoV-2 | Homo sapiens (human) | DDX18 | Q28 | Q9NVP1 | 30 |
| 34672947 | SARS-CoV-2 | Homo sapiens (human) | MAGD2 | Q263 | Q9UNF1 | 25 |
| 34672947 | SARS-CoV-2 | Homo sapiens (human) | PSMD4 | Q27 | P55036 | 17 |
| 34672947 | SARS-CoV-2 | Homo sapiens (human) | HM20A | Q62 | Q9NP66 | 20 |
| 34672947 | SARS-CoV-2 | Homo sapiens (human) | K2C8 | Q70 | P05787 | 27 |
| 34672947 | SARS-CoV-2 | Homo sapiens (human) | PTBP3 | Q152 | O95758 | 27 |
| 34672947 | SARS-CoV-2 | Homo sapiens (human) | PO2F1 | Q238 | P14859 | 78 |
| 34672947 | SARS-CoV-2 | Homo sapiens (human) | CSTF2 | Q279 | P33240 | 45 |
| 34672947 | SARS-CoV-2 | Homo sapiens (human) | ATF1 | Q150 | P18846 | 25 |
| 34672947 | SARS-CoV-2 | Homo sapiens (human) | SIN3A | Q257 | Q96ST3 | 96 |
| 34672947 | SARS-CoV-2 | Homo sapiens (human) | CLN6 | Q26 | Q9NWW5 | 7 |
| 34672947 | SARS-CoV-2 | Homo sapiens (human) | PUF60 | Q99 | Q9UHX1 | 39 |
| 34672947 | SARS-CoV-2 | Homo sapiens (human) | GOGB1 | Q203 | Q14789 | 345 |
| 34672947 | SARS-CoV-2 | Homo sapiens (human) | APBB1 | Q41 | O00213 | 34 |
| 34672947 | SARS-CoV-2 | Homo sapiens (human) | COPRS | Q4 | Q9NQ92 | 11 |
| 34672947 | SARS-CoV-2 | Homo sapiens (human) | SNX6 | Q27 | Q9UNH7 | 16 |
| 34672947 | SARS-CoV-2 | Homo sapiens (human) | BUB1B | Q42 | O60566 | 68 |
| 34672947 | SARS-CoV-2 | Homo sapiens (human) | ARHGG | Q178 | Q5VV41 | 48 |
| 34672947 | SARS-CoV-2 | Homo sapiens (human) | CALD1 | Q668 | Q05682 | 47 |
| 34672947 | SARS-CoV-2 | Homo sapiens (human) | NIPBL | Q298 | Q6KC79 | 153 |
| 34672947 | SARS-CoV-2 | Homo sapiens (human) | PCM1 | Q1823 | Q15154 | 146 |
| 34672947 | SARS-CoV-2 | Homo sapiens (human) | USP9X | Q1199 | Q93008 | 135 |
| 34672947 | SARS-CoV-2 | Homo sapiens (human) | LRC47 | Q132 | Q8N1G4 | 22 |
| 34672947 | SARS-CoV-2 | Homo sapiens (human) | RPP40 | Q223 | O75818 | 11 |
| 34672947 | SARS-CoV-2 | Homo sapiens (human) | MYCT | Q52 | Q96QE2 | 21 |
| 34672947 | SARS-CoV-2 | Homo sapiens (human) | PEG10 | Q31 | Q86TG7 | 36 |
| 34672947 | SARS-CoV-2 | Homo sapiens (human) | RABL6 | Q26 | Q3YEC7 | 34 |
| 34672947 | SARS-CoV-2 | Homo sapiens (human) | TTC4 | Q65 | O95801 | 18 |
| 34672947 | SARS-CoV-2 | Homo sapiens (human) | CDV3 | Q199 | Q9UKY7 | 13 |
| 34672947 | SARS-CoV-2 | Homo sapiens (human) | STRP1 | Q559 | Q5VSL9 | 36 |
| 34672947 | SARS-CoV-2 | Homo sapiens (human) | TWF1 | Q176 | Q12792 | 20 |
| 34672947 | SARS-CoV-2 | Homo sapiens (human) | MYH10 | Q1563 | P35580 | 148 |
| 34672947 | SARS-CoV-2 | Homo sapiens (human) | PTBP2 | Q151 | Q9UKA9 | 29 |
| 34672947 | SARS-CoV-2 | Homo sapiens (human) | KDM2A | Q820 | Q9Y2K7 | 45 |
| 34672947 | SARS-CoV-2 | Homo sapiens (human) | RPA34 | Q88 | O15446 | 36 |
| 34672947 | SARS-CoV-2 | Homo sapiens (human) | SNUT2 | Q241 | Q53GS9 | 23 |
| 34672947 | SARS-CoV-2 | Homo sapiens (human) | TXLNG | Q195 | Q9NUQ3 | 45 |
| 34672947 | SARS-CoV-2 | Homo sapiens (human) | MESH1 | Q56 | Q8N4P3 | 12 |
| 34672947 | SARS-CoV-2 | Homo sapiens (human) | TAB1 | Q444 | Q15750 | 31 |
| 34672947 | SARS-CoV-2 | Homo sapiens (human) | YAP1 | Q133 | P46937 | 51 |
| 34672947 | SARS-CoV-2 | Homo sapiens (human) | LEG8 | Q158 | O00214 | 9 |
| 34672947 | SARS-CoV-2 | Homo sapiens (human) | FYCO1 | Q979 | Q9BQS8 | 157 |
| 34672947 | SARS-CoV-2 | Homo sapiens (human) | EIF3A | Q512 | Q14152 | 68 |
| 34672947 | SARS-CoV-2 | Homo sapiens (human) | ITPR3 | Q903 | Q14573 | 127 |
| 34672947 | SARS-CoV-2 | Homo sapiens (human) | LIMA1 | Q598 | Q9UHB6 | 35 |
| 34672947 | SARS-CoV-2 | Homo sapiens (human) | SMC4 | Q741 | Q9NTJ3 | 63 |
| 34672947 | SARS-CoV-2 | Homo sapiens (human) | VAT1 | Q65 | Q99536 | 14 |
| 34672947 | SARS-CoV-2 | Homo sapiens (human) | AHNK | Q5849 | Q09666 | 68 |
| 34672947 | SARS-CoV-2 | Homo sapiens (human) | LS14A | Q158 | Q8ND56 | 21 |
| 34672947 | SARS-CoV-2 | Homo sapiens (human) | EPMIP | Q141 | Q7L775 | 24 |
| 34672947 | SARS-CoV-2 | Homo sapiens (human) | SMC5 | Q43 | Q8IY18 | 67 |
| 34672947 | SARS-CoV-2 | Homo sapiens (human) | CAPR1 | Q562 | Q14444 | 80 |
| 34672947 | SARS-CoV-2 | Homo sapiens (human) | TPR | Q1322 | P12270 | 220 |
| 34672947 | SARS-CoV-2 | Homo sapiens (human) | APC | Q1338 | P25054 | 154 |
| 34672947 | SARS-CoV-2 | Homo sapiens (human) | IRS2 | Q1122 | Q9Y4H2 | 40 |
| 34672947 | SARS-CoV-2 | Homo sapiens (human) | G45IP | Q157 | Q8TAE8 | 21 |
| 34672947 | SARS-CoV-2 | Homo sapiens (human) | PAR10 | Q288 | Q53GL7 | 63 |
| 34672947 | SARS-CoV-2 | Homo sapiens (human) | T22D2 | M427* | O75157 | 70 |
| 34672947 | SARS-CoV-2 | Homo sapiens (human) | MCM4 | H76* | P33991 | 49 |
| 34672947 | SARS-CoV-2 | Homo sapiens (human) | ZYX | H237* | Q15942 | 45 |
| 34672947 | SARS-CoV-2 | Homo sapiens (human) | R3HD1 | H946* | Q15032 | 106 |
| 34672947 | SARS-CoV-2 | Homo sapiens (human) | CC167 | H37* | Q9P0B6 | 2 |
| 34672947 | SARS-CoV-2 | Homo sapiens (human) | RBBP6 | H481* | Q7Z6E9 | 56 |
| 34672947 | SARS-CoV-2 | Homo sapiens (human) | M4K5 | M456* | Q9Y4K4 | 35 |
| 34672947 | SARS-CoV-2 | Homo sapiens (human) | C2D1A | H273* | Q6P1N0 | 65 |
| 34672947 | SARS-CoV-2 | Homo sapiens (human) | RBM15 | H124* | Q96T37 | 28 |

**Table S5** The prediction performances of the RF model on the test dataset when taking different cutoffs for determining the predicted positive sample.

| **Cutoff** | **TP** | **TN** | **FP** | **FN** | **Accuracy** | **Sensitivity** | **Precision** |
| --- | --- | --- | --- | --- | --- | --- | --- |
| 0.5 | 84 | 5984 | 342 | 21 | 0.944 | 0.800 | 0.197 |
| 0.9 | 62 | 6226 | 100 | 43 | 0.978 | 0.590 | 0.383 |
| 0.99 | 19 | 6305 | 21 | 86 | 0.983 | 0.181 | 0.475 |

TP, true positive; TN, true negative; FP, false positive; FN, false negative.

**Table S6** The functional enrichment analysis of the human proteins which were predicted to be cleaved by coronavirus 3C-like protease.

| **Part I**: KEGG enrichment analysis | | | | | | |
| --- | --- | --- | --- | --- | --- | --- |
| **ID** | **Description** | **Gene Ratio** | **Bg Ratio** | ***P* value** | **p. adjust** | ***q* value** |
| hsa05132 | Salmonella infection | 30/480 | 249/8106 | 0.000148366 | 0.040720013 | 0.040720013 |
| hsa00310 | Lysine degradation | 12/480 | 63/8106 | 0.000270565 | 0.040720013 | 0.040720013 |
| **Part II**: GO enrichment analysis in the domain of Biological Process | | | | | | |
| GO:0007409 | axonogenesis | 55/1208 | 482/18866 | 2.22812E-05 | 0.007237203 | 0.006804553 |
| GO:0022604 | regulation of cell morphogenesis | 55/1208 | 499/18866 | 5.81032E-05 | 0.010784372 | 0.010139667 |
| GO:0044782 | cilium organization | 54/1208 | 397/18866 | 1.24487E-07 | 0.00016174 | 0.000152071 |
| GO:0043087 | regulation of GTPase activity | 54/1208 | 481/18866 | 4.12521E-05 | 0.008699417 | 0.008179353 |
| GO:0007018 | microtubule-based movement | 52/1208 | 368/18866 | 6.17203E-08 | 0.00010692 | 0.000100528 |
| GO:0051656 | establishment of organelle localization | 52/1208 | 432/18866 | 8.49435E-06 | 0.004414513 | 0.004150607 |
| GO:0060271 | cilium assembly | 51/1208 | 377/18866 | 3.29625E-07 | 0.000342613 | 0.000322131 |
| GO:0048193 | Golgi vesicle transport | 50/1208 | 374/18866 | 6.14703E-07 | 0.000532435 | 0.000500605 |
| GO:0048285 | organelle fission | 49/1208 | 476/18866 | 0.00070084 | 0.044713086 | 0.042040073 |
| GO:1901990 | regulation of mitotic cell cycle phase transition | 47/1208 | 448/18866 | 0.000593755 | 0.04060189 | 0.03817465 |
| GO:0043547 | positive regulation of GTPase activity | 45/1208 | 406/18866 | 0.000234234 | 0.02628391 | 0.02471262 |
| GO:0000280 | nuclear division | 45/1208 | 428/18866 | 0.000734094 | 0.045417711 | 0.042702574 |
| GO:0032535 | regulation of cellular component size | 44/1208 | 383/18866 | 0.00012261 | 0.018205822 | 0.017117451 |
| GO:0030900 | forebrain development | 44/1208 | 391/18866 | 0.00019552 | 0.024193319 | 0.022747007 |
| GO:0031346 | positive regulation of cell projection organization | 44/1208 | 394/18866 | 0.000231675 | 0.02628391 | 0.02471262 |
| GO:0006913 | nucleocytoplasmic transport | 43/1208 | 354/18866 | 4.03237E-05 | 0.008699417 | 0.008179353 |
| GO:0051169 | nuclear transport | 43/1208 | 357/18866 | 4.92238E-05 | 0.009474678 | 0.008908268 |
| GO:0048880 | sensory system development | 43/1208 | 394/18866 | 0.000442968 | 0.032895709 | 0.030929155 |
| GO:1902903 | regulation of supramolecular fiber organization | 42/1208 | 373/18866 | 0.000266921 | 0.027151579 | 0.025528418 |
| GO:0150063 | visual system development | 42/1208 | 388/18866 | 0.000606666 | 0.040946004 | 0.038498192 |
| GO:0010769 | regulation of cell morphogenesis involved in differentiation | 39/1208 | 310/18866 | 4.18483E-05 | 0.008699417 | 0.008179353 |
| GO:0051258 | protein polymerization | 37/1208 | 300/18866 | 9.88908E-05 | 0.016060492 | 0.015100372 |
| GO:0010976 | positive regulation of neuron projection development | 36/1208 | 290/18866 | 0.000106951 | 0.01684311 | 0.015836204 |
| GO:0030705 | cytoskeleton-dependent intracellular transport | 35/1208 | 200/18866 | 4.97466E-08 | 0.00010692 | 0.000100528 |
| GO:0007411 | axon guidance | 34/1208 | 284/18866 | 0.000326136 | 0.028909548 | 0.027181293 |
| GO:0097485 | neuron projection guidance | 34/1208 | 285/18866 | 0.000348113 | 0.028909548 | 0.027181293 |
| GO:0051648 | vesicle localization | 33/1208 | 229/18866 | 1.02185E-05 | 0.00482778 | 0.004539168 |
| GO:0051650 | establishment of vesicle localization | 31/1208 | 212/18866 | 1.39599E-05 | 0.005580742 | 0.005247117 |
| GO:0007163 | establishment or maintenance of cell polarity | 31/1208 | 220/18866 | 2.92914E-05 | 0.007248917 | 0.006815567 |
| GO:0060560 | developmental growth involved in morphogenesis | 31/1208 | 236/18866 | 0.000112356 | 0.017173949 | 0.016147266 |
| GO:0099111 | microtubule-based transport | 30/1208 | 190/18866 | 4.04187E-06 | 0.002625701 | 0.002468733 |
| GO:0006888 | endoplasmic reticulum to Golgi vesicle-mediated transport | 30/1208 | 206/18866 | 2.06835E-05 | 0.007166145 | 0.006737742 |
| GO:0032271 | regulation of protein polymerization | 30/1208 | 231/18866 | 0.000176349 | 0.022353293 | 0.02101698 |
| GO:0048588 | developmental cell growth | 30/1208 | 237/18866 | 0.000276942 | 0.02715595 | 0.025532528 |
| GO:1902749 | regulation of cell cycle G2/M phase transition | 27/1208 | 217/18866 | 0.000714102 | 0.044713086 | 0.042040073 |
| GO:0010389 | regulation of G2/M transition of mitotic cell cycle | 26/1208 | 200/18866 | 0.000455693 | 0.032895709 | 0.030929155 |
| GO:0017038 | protein import | 26/1208 | 200/18866 | 0.000455693 | 0.032895709 | 0.030929155 |
| GO:0010970 | transport along microtubule | 25/1208 | 161/18866 | 3.34096E-05 | 0.007892263 | 0.007420452 |
| GO:0051170 | import into nucleus | 25/1208 | 171/18866 | 9.19735E-05 | 0.015932874 | 0.014980383 |
| GO:0050770 | regulation of axonogenesis | 25/1208 | 186/18866 | 0.000350402 | 0.028909548 | 0.027181293 |
| GO:0031023 | microtubule organizing center organization | 24/1208 | 136/18866 | 5.26371E-06 | 0.003039502 | 0.002857796 |
| GO:0006606 | protein import into nucleus | 24/1208 | 150/18866 | 2.89067E-05 | 0.007248917 | 0.006815567 |
| GO:0140056 | organelle localization by membrane tethering | 24/1208 | 171/18866 | 0.000237703 | 0.02628391 | 0.02471262 |
| GO:0022406 | membrane docking | 24/1208 | 180/18866 | 0.000512855 | 0.036511083 | 0.034328398 |
| GO:0010770 | positive regulation of cell morphogenesis involved in differentiation | 23/1208 | 157/18866 | 0.000167424 | 0.022074047 | 0.020754429 |
| GO:0021915 | neural tube development | 23/1208 | 163/18866 | 0.000293435 | 0.028003166 | 0.026329096 |
| GO:0007156 | homophilic cell adhesion via plasma membrane adhesion molecules | 23/1208 | 168/18866 | 0.000455742 | 0.032895709 | 0.030929155 |
| GO:1990138 | neuron projection extension | 23/1208 | 172/18866 | 0.000637405 | 0.04193155 | 0.039424821 |
| GO:0007098 | centrosome cycle | 22/1208 | 125/18866 | 1.34008E-05 | 0.005580742 | 0.005247117 |
| GO:0048813 | dendrite morphogenesis | 22/1208 | 146/18866 | 0.000151966 | 0.020853039 | 0.019606414 |
| GO:0007030 | Golgi organization | 22/1208 | 151/18866 | 0.000248911 | 0.026592598 | 0.025002854 |
| GO:0030010 | establishment of cell polarity | 21/1208 | 141/18866 | 0.000250729 | 0.026592598 | 0.025002854 |
| GO:1902850 | microtubule cytoskeleton organization involved in mitosis | 21/1208 | 144/18866 | 0.000336326 | 0.028909548 | 0.027181293 |
| GO:0016571 | histone methylation | 20/1208 | 143/18866 | 0.000793956 | 0.048543422 | 0.045641426 |
| GO:0099518 | vesicle cytoskeletal trafficking | 18/1208 | 75/18866 | 8.38431E-07 | 0.000622475 | 0.000585262 |
| GO:0097711 | ciliary basal body-plasma membrane docking | 18/1208 | 95/18866 | 2.87899E-05 | 0.007248917 | 0.006815567 |
| GO:0050830 | defense response to Gram-positive bacterium | 17/1208 | 99/18866 | 0.000169898 | 0.022074047 | 0.020754429 |
| GO:0072384 | organelle transport along microtubule | 15/1208 | 85/18866 | 0.000296358 | 0.028003166 | 0.026329096 |
| GO:0001843 | neural tube closure | 15/1208 | 91/18866 | 0.000630717 | 0.04193155 | 0.039424821 |
| GO:0060606 | tube closure | 15/1208 | 92/18866 | 0.000709813 | 0.044713086 | 0.042040073 |
| GO:0051298 | centrosome duplication | 14/1208 | 68/18866 | 8.53026E-05 | 0.015286807 | 0.01437294 |
| GO:0046785 | microtubule polymerization | 14/1208 | 77/18866 | 0.000339306 | 0.028909548 | 0.027181293 |
| GO:0008088 | axo-dendritic transport | 13/1208 | 70/18866 | 0.000441924 | 0.032895709 | 0.030929155 |
| GO:0033047 | regulation of mitotic sister chromatid segregation | 13/1208 | 72/18866 | 0.00058623 | 0.04060189 | 0.03817465 |
| GO:0047496 | vesicle transport along microtubule | 12/1208 | 49/18866 | 4.53148E-05 | 0.009057734 | 0.00851625 |
| GO:0098930 | axonal transport | 12/1208 | 59/18866 | 0.000303759 | 0.028189957 | 0.026504721 |
| GO:0006607 | NLS-bearing protein import into nucleus | 11/1208 | 20/18866 | 6.96515E-09 | 3.61979E-05 | 3.40339E-05 |
| GO:0051653 | spindle localization | 11/1208 | 50/18866 | 0.000261277 | 0.027151579 | 0.025528418 |
| GO:0051293 | establishment of spindle localization | 10/1208 | 45/18866 | 0.00045326 | 0.032895709 | 0.030929155 |
| GO:0035459 | vesicle cargo loading | 9/1208 | 27/18866 | 2.87055E-05 | 0.007248917 | 0.006815567 |
| GO:0061842 | microtubule organizing center localization | 9/1208 | 31/18866 | 9.78332E-05 | 0.016060492 | 0.015100372 |
| GO:0040001 | establishment of mitotic spindle localization | 9/1208 | 35/18866 | 0.000271673 | 0.027151579 | 0.025528418 |
| GO:0008090 | retrograde axonal transport | 8/1208 | 20/18866 | 1.7367E-05 | 0.006446876 | 0.006061472 |
| GO:0042832 | defense response to protozoan | 8/1208 | 26/18866 | 0.000152476 | 0.020853039 | 0.019606414 |
| GO:0001562 | response to protozoan | 8/1208 | 27/18866 | 0.000204589 | 0.024348329 | 0.02289275 |
| GO:0000132 | establishment of mitotic spindle orientation | 8/1208 | 30/18866 | 0.000454191 | 0.032895709 | 0.030929155 |
| GO:0051642 | centrosome localization | 8/1208 | 30/18866 | 0.000454191 | 0.032895709 | 0.030929155 |
| GO:0051673 | membrane disruption in other organism | 6/1208 | 11/18866 | 2.37802E-05 | 0.007248917 | 0.006815567 |
| GO:0090161 | Golgi ribbon formation | 6/1208 | 14/18866 | 0.00013076 | 0.018876703 | 0.017748227 |
| GO:0032252 | secretory granule localization | 6/1208 | 15/18866 | 0.000206143 | 0.024348329 | 0.02289275 |
| GO:0007221 | positive regulation of transcription of Notch receptor target | 6/1208 | 18/18866 | 0.000647384 | 0.042055664 | 0.039541515 |
| GO:0032253 | dense core granule localization | 5/1208 | 11/18866 | 0.000356015 | 0.028909548 | 0.027181293 |
| GO:0099519 | dense core granule cytoskeletal transport | 5/1208 | 11/18866 | 0.000356015 | 0.028909548 | 0.027181293 |
| GO:1901950 | dense core granule transport | 5/1208 | 11/18866 | 0.000356015 | 0.028909548 | 0.027181293 |
| GO:1902287 | semaphorin-plexin signaling pathway involved in axon guidance | 5/1208 | 12/18866 | 0.000578185 | 0.04060189 | 0.03817465 |
| **Part III**: GO enrichment analysis in the domain of Cellular Component | | | | | | |
| GO:0005874 | microtubule | 56/1260 | 431/19559 | 4.19579E-07 | 5.42935E-05 | 4.65512E-05 |
| GO:0005635 | nuclear envelope | 46/1260 | 473/19559 | 0.003505494 | 0.046286835 | 0.039686264 |
| GO:0005938 | cell cortex | 43/1260 | 305/19559 | 1.06602E-06 | 0.000114952 | 9.85599E-05 |
| GO:0098687 | chromosomal region | 43/1260 | 350/19559 | 3.56793E-05 | 0.001648895 | 0.00141376 |
| GO:0016607 | nuclear speck | 43/1260 | 401/19559 | 0.000725213 | 0.016757604 | 0.014367945 |
| GO:0005819 | spindle | 42/1260 | 367/19559 | 0.00021538 | 0.00696753 | 0.00597395 |
| GO:0099568 | cytoplasmic region | 39/1260 | 254/19559 | 3.79449E-07 | 5.42935E-05 | 4.65512E-05 |
| GO:0150034 | distal axon | 39/1260 | 309/19559 | 4.47018E-05 | 0.001928138 | 0.001653183 |
| GO:0030427 | site of polarized growth | 33/1260 | 191/19559 | 1.88409E-07 | 5.31583E-05 | 4.55779E-05 |
| GO:0043292 | contractile fiber | 33/1260 | 238/19559 | 2.60684E-05 | 0.001297402 | 0.001112391 |
| GO:0030426 | growth cone | 32/1260 | 184/19559 | 2.46484E-07 | 5.31583E-05 | 4.55779E-05 |
| GO:0030016 | myofibril | 32/1260 | 227/19559 | 2.46113E-05 | 0.001297402 | 0.001112391 |
| GO:0032838 | plasma membrane bounded cell projection cytoplasm | 31/1260 | 211/19559 | 1.43511E-05 | 0.000844107 | 0.000723736 |
| GO:0000793 | condensed chromosome | 28/1260 | 222/19559 | 0.000509589 | 0.012680927 | 0.010872608 |
| GO:0000922 | spindle pole | 27/1260 | 165/19559 | 6.87595E-06 | 0.000494305 | 0.000423816 |
| GO:0030017 | sarcomere | 26/1260 | 207/19559 | 0.000844257 | 0.017620467 | 0.015107763 |
| GO:0098858 | actin-based cell projection | 26/1260 | 220/19559 | 0.002043717 | 0.030038925 | 0.02575533 |
| GO:0000775 | chromosome, centromeric region | 25/1260 | 196/19559 | 0.000841354 | 0.017620467 | 0.015107763 |
| GO:0030863 | cortical cytoskeleton | 23/1260 | 106/19559 | 2.12494E-07 | 5.31583E-05 | 4.55779E-05 |
| GO:0019814 | immunoglobulin complex | 23/1260 | 163/19559 | 0.000320667 | 0.009204684 | 0.007892083 |
| GO:0030496 | midbody | 23/1260 | 182/19559 | 0.001506085 | 0.024360929 | 0.020887024 |
| GO:0005814 | centriole | 22/1260 | 143/19559 | 0.000121932 | 0.004930624 | 0.00422751 |
| GO:0005875 | microtubule associated complex | 22/1260 | 159/19559 | 0.000563058 | 0.013492548 | 0.011568491 |
| GO:0072686 | mitotic spindle | 19/1260 | 133/19559 | 0.000880645 | 0.017805541 | 0.015266445 |
| GO:0072562 | blood microparticle | 19/1260 | 148/19559 | 0.003111335 | 0.041938206 | 0.035957756 |
| GO:0005643 | nuclear pore | 18/1260 | 85/19559 | 6.26483E-06 | 0.000494305 | 0.000423816 |
| GO:0005930 | axoneme | 18/1260 | 122/19559 | 0.00081151 | 0.017620467 | 0.015107763 |
| GO:0097014 | ciliary plasm | 18/1260 | 124/19559 | 0.000984846 | 0.018741031 | 0.016068532 |
| GO:0030864 | cortical actin cytoskeleton | 15/1260 | 80/19559 | 0.000158066 | 0.006015792 | 0.005157931 |
| GO:0034708 | methyltransferase complex | 15/1260 | 105/19559 | 0.002944146 | 0.04052899 | 0.034749496 |
| GO:0016459 | myosin complex | 14/1260 | 57/19559 | 1.10678E-05 | 0.000716084 | 0.000613969 |
| GO:0031594 | neuromuscular junction | 13/1260 | 76/19559 | 0.001054027 | 0.019484436 | 0.016705927 |
| GO:0035097 | histone methyltransferase complex | 13/1260 | 77/19559 | 0.001194737 | 0.020891756 | 0.01791256 |
| GO:0120111 | neuron projection cytoplasm | 13/1260 | 87/19559 | 0.003671867 | 0.047513955 | 0.040738395 |
| GO:1904115 | axon cytoplasm | 12/1260 | 57/19559 | 0.000228788 | 0.007048844 | 0.006043668 |
| GO:0070971 | endoplasmic reticulum exit site | 11/1260 | 33/19559 | 3.92788E-06 | 0.000363049 | 0.000311277 |
| GO:0016328 | lateral plasma membrane | 11/1260 | 65/19559 | 0.00272677 | 0.038352617 | 0.032883477 |
| GO:0005871 | kinesin complex | 10/1260 | 51/19559 | 0.00134117 | 0.02224966 | 0.019076826 |
| GO:0030286 | dynein complex | 10/1260 | 53/19559 | 0.001820959 | 0.028735622 | 0.02463788 |
| GO:0097431 | mitotic spindle pole | 8/1260 | 30/19559 | 0.000473329 | 0.012249752 | 0.010502919 |
| GO:1990752 | microtubule end | 8/1260 | 34/19559 | 0.001166733 | 0.020891756 | 0.01791256 |
| GO:0031672 | A band | 8/1260 | 37/19559 | 0.002089261 | 0.030038925 | 0.02575533 |
| GO:0032982 | myosin filament | 7/1260 | 22/19559 | 0.000327214 | 0.009204684 | 0.007892083 |
| GO:0044665 | MLL1/2 complex | 7/1260 | 29/19559 | 0.002014218 | 0.030038925 | 0.02575533 |
| GO:0071339 | MLL1 complex | 7/1260 | 29/19559 | 0.002014218 | 0.030038925 | 0.02575533 |
| GO:0005859 | muscle myosin complex | 6/1260 | 15/19559 | 0.000213205 | 0.00696753 | 0.00597395 |
| GO:0098831 | presynaptic active zone cytoplasmic component | 6/1260 | 15/19559 | 0.000213205 | 0.00696753 | 0.00597395 |
| GO:0043034 | costamere | 6/1260 | 19/19559 | 0.000924617 | 0.018128107 | 0.015543012 |
| GO:0016460 | myosin II complex | 6/1260 | 20/19559 | 0.001249451 | 0.021273545 | 0.018239906 |
| GO:0002116 | semaphorin receptor complex | 5/1260 | 11/19559 | 0.000366342 | 0.009875969 | 0.008467641 |
| **Part IV**: GO enrichment analysis in the domain of Molecular Function | | | | | | |
| GO:0017016 | Ras GTPase binding | 58/1221 | 415/18352 | 6.05046E-08 | 1.82926E-05 | 1.74083E-05 |
| GO:0016887 | ATPase activity | 58/1221 | 423/18352 | 1.18241E-07 | 2.68112E-05 | 2.55152E-05 |
| GO:0031267 | small GTPase binding | 58/1221 | 428/18352 | 1.77547E-07 | 3.22071E-05 | 3.06503E-05 |
| GO:0003779 | actin binding | 56/1221 | 437/18352 | 1.76087E-06 | 0.000177457 | 0.000168879 |
| GO:0015631 | tubulin binding | 47/1221 | 365/18352 | 1.03403E-05 | 0.000586168 | 0.000557834 |
| GO:0060589 | nucleoside-triphosphatase regulator activity | 42/1221 | 348/18352 | 0.000133131 | 0.005249973 | 0.004996202 |
| GO:0030695 | GTPase regulator activity | 41/1221 | 307/18352 | 1.57167E-05 | 0.000791948 | 0.000753667 |
| GO:0008017 | microtubule binding | 38/1221 | 265/18352 | 6.15841E-06 | 0.000430919 | 0.000410089 |
| GO:0005085 | guanyl-nucleotide exchange factor activity | 36/1221 | 215/18352 | 2.60565E-07 | 3.93887E-05 | 3.74848E-05 |
| GO:0005096 | GTPase activator activity | 35/1221 | 275/18352 | 0.000168825 | 0.006380193 | 0.00607179 |
| GO:0051015 | actin filament binding | 32/1221 | 206/18352 | 6.17635E-06 | 0.000430919 | 0.000410089 |
| GO:0003774 | motor activity | 31/1221 | 132/18352 | 4.67844E-10 | 4.24335E-07 | 4.03823E-07 |
| GO:0005516 | calmodulin binding | 30/1221 | 200/18352 | 2.38203E-05 | 0.001137106 | 0.001082141 |
| GO:0005088 | Ras guanyl-nucleotide exchange factor activity | 23/1221 | 115/18352 | 1.69792E-06 | 0.000177457 | 0.000168879 |
| GO:0005200 | structural constituent of cytoskeleton | 19/1221 | 104/18352 | 5.00686E-05 | 0.002162486 | 0.002057957 |
| GO:0003777 | microtubule motor activity | 18/1221 | 77/18352 | 2.18945E-06 | 0.000198583 | 0.000188984 |
| GO:1990939 | ATP-dependent microtubule motor activity | 14/1221 | 34/18352 | 1.22024E-08 | 5.53377E-06 | 5.26628E-06 |
| GO:0008536 | Ran GTPase binding | 12/1221 | 41/18352 | 9.23494E-06 | 0.000558406 | 0.000531414 |
| GO:0051959 | dynein light intermediate chain binding | 11/1221 | 27/18352 | 5.22619E-07 | 6.77165E-05 | 6.44432E-05 |
| GO:0008139 | nuclear localization sequence binding | 10/1221 | 27/18352 | 4.85749E-06 | 0.000400522 | 0.000381162 |
| GO:0045505 | dynein intermediate chain binding | 10/1221 | 30/18352 | 1.43938E-05 | 0.00076795 | 0.000730829 |
| GO:0017112 | Rab guanyl-nucleotide exchange factor activity | 10/1221 | 33/18352 | 3.69077E-05 | 0.001673765 | 0.001592859 |
| GO:0005048 | signal sequence binding | 10/1221 | 49/18352 | 0.001238669 | 0.040124041 | 0.038184545 |
| GO:0000146 | microfilament motor activity | 9/1221 | 29/18352 | 7.35726E-05 | 0.003033197 | 0.00288658 |
| GO:0008569 | ATP-dependent microtubule motor activity, minus-end-directed | 8/1221 | 18/18352 | 8.99164E-06 | 0.000558406 | 0.000531414 |
| GO:0008574 | ATP-dependent microtubule motor activity, plus-end-directed | 6/1221 | 16/18352 | 0.00038405 | 0.013933333 | 0.013259831 |
| GO:0098918 | structural constituent of synapse | 6/1221 | 18/18352 | 0.000793367 | 0.026651255 | 0.025363 |
| GO:0061608 | nuclear import signal receptor activity | 6/1221 | 20/18352 | 0.001476619 | 0.046182521 | 0.043950174 |
| GO:0017154 | semaphorin receptor activity | 5/1221 | 12/18352 | 0.0006898 | 0.024063393 | 0.022900228 |
